# SG-APSIC1209: Risk factors associated with mortality among carbapenem-resistant Enterobacteriaceae inpatient in a tertiary-care teaching hospital in Malaysia

**DOI:** 10.1017/ash.2023.75

**Published:** 2023-03-16

**Authors:** Sasheela Sri La Sri Ponnampalavanar, Arulvani Rajandra, Nur Alwani Suhaimi, Cindy Teh Shuan Ji, Sia Jia Xuen, Tan Shu Fan, Siti Zuhairah binti Mohd Razali, Kam Yit Yin, Zhi Xian Kong, Min Yi Lau, Yee Qing Lee, Siti Norintan Zainon, Anjanna Kukreja, Suzana Saaibon, Siti Shuhaida Shamsudin

**Affiliations:** University Malaya Medical Centre, Malaysia; University Malaya Medical Centre, Malaysia; University Malaya Medical Centre, Malaysia; University Malaya Medical Centre, Malaysia; University Malaya Medical Centre, Malaysia; University Malaya Medical Centre, Malaysia; University Malaya Medical Centre, Malaysia; University Malaya Medical Centre, Malaysia; University Malaya Medical Centre, Malaysia; University Malaya Medical Centre, Malaysia; University Malaya Medical Centre, Malaysia; University Malaya Medical Centre, Malaysia; University Malaya Medical Centre, Malaysia; University Malaya Medical Centre, Malaysia; University Malaya Medical Centre, Malaysia

## Abstract

**Objectives:** Carbapenem-resistant Enterobacteriaceae (CRE) is a multidrug-resistant gram-negative bacteria (MDR-GNB) that is rapidly emerging as a life-threatening nosocomial disease in many countries. We sought to identify the risk factors associated with mortality for carriage of CRE in patients at a tertiary-care teaching hospital. **Methods:** A retrospective observational study was conducted between January 2020 to December 2021 in a tertiary-care teaching healthcare facility, University Malaya Medical Centre in Malaysia. The study included all inpatients aged ≥18 years who had a CRE infection or were colonized during the study period. The genotype was identified by polymerase chain reaction (PCR). Statistical analysis of data including a multivariate logistic regression analysis was conducted using SPSS version 23.0 software. **Results:** In total, 176 cases of CRE (130 infection and 46 colonized) were identified, and the mortality rate was 31.8%. The main sources of CRE were rectal swab (61.9%), blood (11.9%), and respiratory sources (11.9%). *Klebsiella pneumoniae* (55.7%) was the predominant species, followed by *Escherichia coli* (21.6%). Among the isolates, 17.7% were non–CPE-CRE and 82.3% were CPE-CRE: NDM (69.3%) and OXA (10.8%). In multivariate analysis, the factors associated with mortality were older age (OR, 1.040; 95% CI, 1.012–1.069), longer length of stay (OR, 0.974; 95% CI, 0.955–0.994), use of central venous catheter (OR, 0.287; 95% CI, 0.094–0.878), and arterial lines (OR, 0.292; 95% CI, 0.095–0.891). **Conclusion:** Patients with CRE had a high mortality rate. Older age, longer duration of stay, indwelling CVC and arterial line were independent risk factors for death. Infection prevention and control measures to reduce CRE, such as active surveillance, contact precautions, compliance to intravenous catheter care bundles, healthcare worker education, and hand hygiene adherence, should be implemented.

